# A Comparison of Hospital Expenditure in 1913 with 1912

**Published:** 1914-10-03

**Authors:** 


### KING EDWARD'S HOSPITAL FUND FOR LONDON.

### A Comparison of Hospital Expenditure in 1913 with 1912.

THE following table gives a comparison of the expenditure at one hundred and one hospitals in 1912 and 1913:—

| London Hospitals | | Beds Occupied | Average Cost per Bed | Out-Patient Attendances | Average Cost per Attendance | Total Value of Differences in Cost | | Net Difference |
|---|---|---|---|---|---|---|---|---|
| | | | | | | In-Patients | Out-Patients | |
| | | | £ s. d. | | Pence | £ | £ | £ |
| 15 Larger General ... | 1912 | 4,105 | 81 6 9 | 2,706,275 | 7·29 | +10,513 | + 9,453 | +19,966 |
| | 1913 | 4,120 | 83 17 9 | 2,428,608 | 8·22 | | | |
| 15 Smaller General ... | 19 2 | 771 | 74 13 11 | 621,101 | 5·54 | + 1,656 | + 2,198 | + 3,854 |
| | 1913 | 791 | 76 15 9 | 560,211 | 6·48 | | | |
| 3 Consumption ... | 1912 | 544 | 61 0 10 | 111,971 | 11·84 | + 3,163 | + 1,297 | + 4,460 |
| | 1913 | 543 | 66 17 4 | 84,749 | 15·50 | | | |
| 5 Hospitals for Women ... ... | 1912 | 260 | 77 13 0 | 80,383 | 7·91 | + 560 | + 58 | + 618 |
| | 1913 | 260 | 79 16 1 | 76,826 | 8·09 | | | |
| 6 Children's ... ... | 1912 | 602 | 67 18 11 | 363,258 | 7·47 | − 570 | − 259 | − 829 |
| | 1913 | 620 | 67 0 6 | 399,326 | 7·31 | | | |
| 5 Ophthalmic ... | 1912 | 204 | 56 10 11 | 285,806 | 5·45 | + 359 | + 45 | + 404 |
| | 1913 | 210 | 58 5 1 | 283,656 | 5·49 | | | |
| 3 Hospitals for Epilepsy, etc. ... | 1912 | 240 | 70 13 2 | 92,160 | 8·29 | − 498 | + 54 | − 444 |
| | 1913 | 258 | 68 14 6 | 89,819 | 8·44 | | | |
| 12 Cottage ... ... | 1912 | 192 | 68 6 4 | 30,394 | 3·80 | − 4 | + 95 | + 91 |
| | 1913 | 197 | 68 5 11 | 26,459 | 4·65 | | | |
| 7 Lying-in ... ... | 1912 | 208 | 63 15 10 | 94,267 | 9·35 | + 362 | − 269 | + 93 |
| | 1913 | 192 | 65 13 6 | 68,995 | 8·42 | | | |
| 30 Unclassified ... | 1912 | 1,334 | — | 456,224 | — | − 1,733 | + 1,099 | − 634 |
| | 1213 | 1,379 | — | 423,642 | — | | | |
| | | | | | Net Totals ... | +13,808 | +13,771 | +27,579 |

The above figures are taken from page 11 of the annual "Statistical Report on the ordinary expenditure of one hundred and six London hospitals for the year 1913," which has been published by the authority of the Fund—an invaluable document with which we hope to deal fully in a subsequent issue.

